# Fusiform Gyrus is Related to Subclinical Social Anxiety in Healthy Individuals

**DOI:** 10.1192/j.eurpsy.2023.451

**Published:** 2023-07-19

**Authors:** B. Kim, S.-H. Lee, H.-J. Kim, M.-K. Kim

**Affiliations:** ^1^ CHA Bundang medical center; ^2^CHA Ilsan medical center, Seongnam-si, Gyeonggi-do, Korea, Republic Of

## Abstract

**Introduction:**

Subclinical or subthreshold social anxiety (SSA) is associated with significant burden. Up to 20% of general population report subclinical social anxiety symptoms, which can change individual social, work functioning.

**Objectives:**

However, neural mechanisms of SSA have not been fully investigated in healthy individual yet. This study aimed to examine the relationship between gray matter volumes (GMVs) and SSA.

**Methods:**

We enrolled a total of 57 healthy individuals with SSA. The General Anxiety Disorder-7 (GAD-7), Beck Depression Inventory-II (BDI-II), Beck Anxiety Inventory (BAI), and Albany Panic and Phobia Scale (APPQ) were evaluated. Freesurfer was applied to investigated the relationship between SSA and GMVs. Multiple regression models with age, sex, and total intracranial volume as covariates were performed. Pearson correlation analyses also investigated the exploratory correlations between the GMVs of the SSA-related regions and other psychological characteristics among healthy individuals.

**Results:**

Freesurfer voxel-wise correlational analyses showed a significant negative correlation between the SA scores of APPQ and gray matter volumes (GMVs) in the fusiform gyrus (FG). In addition, the GMVs in the FG were significantly negatively associated with the total GAD-7, BDI-II, BAI, and APPQ scores. Performance anxiety was significantly correlated with posterior cingulate gyrus, parahippocampal gyrus and fusiform gyrus.

**Image:**

**Figure:**
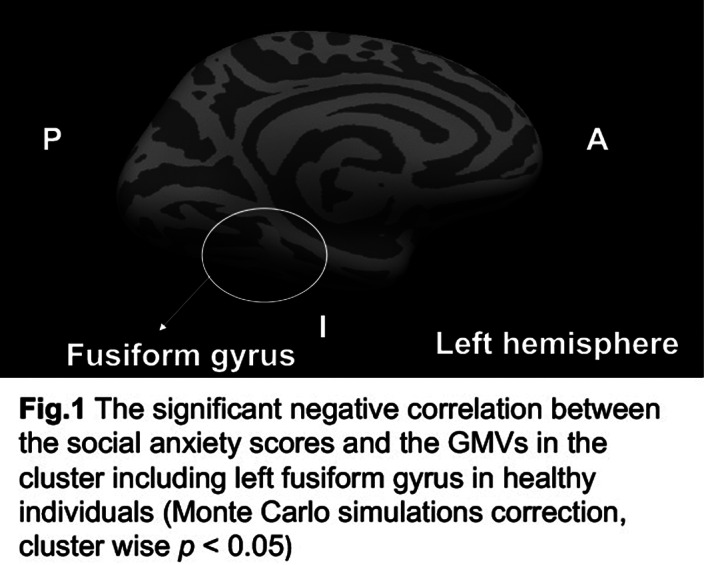

**Conclusions:**

Our findings suggest that healthy individuals with SSA showed decreased GMVs in the FG and the GMVs of FG were associated with general anxiety and depression symptomatology.

**Disclosure of Interest:**

None Declared

